# Cost-effectiveness evaluation based on two models of first-line atezolizumab monotherapy and chemotherapy for advanced non-small cell lung cancer with high-PDL1 expression

**DOI:** 10.3389/fonc.2023.1093469

**Published:** 2023-03-14

**Authors:** Chuan Zhang, Yue Liu, Jing Tan, Panwen Tian, Weimin Li

**Affiliations:** ^1^ Department of Pharmacy, West China Second University Hospital, Sichuan University, Chengdu, China; ^2^ Evidence-Based Pharmacy Center, West China Second University Hospital, Sichuan University, Chengdu, China; ^3^ Key Laboratory of Birth Defects and Related Diseases of Women and Children, Ministry of Education, Sichuan University, Chengdu, China; ^4^ West China School of Pharmacy, Sichuan University, Chengdu, China; ^5^ Chinese Evidence-based Medicine Center, West China Hospital, Sichuan University, Chengdu, China; ^6^ Department of Respiratory, West China Hospital, Sichuan University, Chengdu, China

**Keywords:** atezolizumab, non-small-cell lung cancer, partitioned survival model, Markov model, cost-effectiveness

## Abstract

**Background:**

Atezolizumab may provide clinical benefits to patients with advanced non-small cell lung cancer (NSCLC). However, the price of atezolizumab is relatively high, and its economic outcomes have remained unclear. In this study, we used two models to examine the cost-effectiveness of initial atezolizumab monotherapy versus chemotherapy for patients with PD-L1 high-expressing EGFR and ALK wild-type advanced NSCLC in the context of the Chinese healthcare system.

**Methods:**

Partitioned Survival model and Markov model were performed to evaluate the cost-effectiveness of first-line single-agent atezolizumab versus platinum-based chemotherapy for patients with advanced NSCLC with PD-L1 high-expressing EGFR and ALK wild-type disease. Clinical outcomes and safety information were obtained from the most recent data from the IMpower110 trial, while cost and utility values were obtained from Chinese hospitals and relevant literature. Total costs, life years (LYs), quality-adjusted life years (QALYs), and incremental cost-effectiveness ratios (ICERs) were estimated. One-way and probabilistic sensitivity analyses were performed to explore model uncertainty. Scenario analyses were also conducted for the Patient Assistance Program (PAP) and various provinces in China.

**Results:**

In the Partitioned Survival model, the total cost of atezolizumab was $145,038, providing 2.92 LYs and 2.39 QALYs, while the total cost of chemotherapy was $69,803, providing 2.12 LYs and 1.65 QALYs. The ICER for atezolizumab versus chemotherapy was $102,424.83/QALY; in the Markov model, the ICER was $104,806.71/QALY. Atezolizumab was not cost-effective at the WTP threshold of three times China’s per capita gross domestic product (GDP). Sensitivity analysis showed that the cost of atezolizumab, the utility of PFS, and the discount rate had a significant impact on ICER; PAP significantly reduced ICER, but atezolizumab was still not cost-effective in China.

**Conclusion:**

First-line monotherapy with atezolizumab for patients with PD-L1 high-expressing EGFR and ALK wild-type advanced NSCLC was estimated to be less cost-effective than chemotherapy in terms of the Chinese healthcare system; offering PAP increased the likelihood that atezolizumab would be cost-effective. In some areas of China with higher levels of economic development, atezolizumab was likely to be cost-effective. To improve the cost-effectiveness of atezolizumab, drug prices would need to be reduced.

## Introduction

Lung cancer is the leading cause of global mortality among malignant tumors, with 2.2 million new cases and 1.8 million deaths worldwide in 2020, according to WHO/IARC (1). In China, lung cancer was the leading cause of cancer-related deaths in 2016, leading to approximately 828,000 new cases and 657,000 deaths (2). Lung cancer is histologically classified into small cell lung cancer (SCLC) and non-small cell lung cancer (NSCLC); NSCLC accounts for about 85% of cases (3). Due to the lack of reliable markers for early diagnosis, approximately 60% or more were diagnosed as stages III and IV (4, 5), with 5-year overall survival rates of 15% to 20% (6). The median overall survival of conventional platinum-based two-drug regimens for patients with advanced NSCLC with wild-type driver genes is less than one year, and there is a need to improve their efficacy (7). In recent years, immune checkpoint inhibitors (ICIs) have made tremendous strides in tumor therapy, Multiple studies have shown the efficacy and safety of ICIs to be superior to conventional chemotherapy (8, 9). The NCCN recommends atezolizumab monotherapy as first-line treatment for advanced NSCLC in 2022, based on clinical evidence (10). The IMpower110 was a phase III, a multicenter, randomized, open-controlled trial conducted at 142 institutions in 19 countries (11). Subjects were randomized to atezolizumab and chemotherapy in a 1:1 ratio for each. Patients were divided into high-expressing and high- or intermediate-expressing groups according to PD-L1 expression, and treatment response was compared separately. On July 12, 2021, IMpower110 updated additional 17-month follow-up data, showing that the OS benefit was maintained in the high-expressing group [hazard ration (HR) = 0.76, 95% confidence interval (CI) = 0.54-1.09, median = 20.2 months vs. 14.7 months] (12). Atezolizumab monotherapy has been approved by the Food and Drug Administration (FDA) as first-line therapy for high PD-L1 metastatic NSCLC. In China, the National Medical Products Administration (NMPA) has approved several atezolizumab-related therapies for NSCLC.

Atezolizumab provides clinical benefit but at a relatively high cost. Based on preliminary data from IMpower110, only a few literature studies have examined the cost-effectiveness of first-line atezolizumab monotherapy for advanced NSCLC from a Chinese perspective (13, 14). IMpower110 was updated on July 12, 2021, and no economic studies based on new survival data have been conducted since. Therefore, to explore the cost-effectiveness of first-line single-agent atezolizumab chemotherapy for patients with PD-L1 high-expressing EGFR and ALK wild-type advanced NSCLC, we performed an economic study based on the updated IMpower110 from the perspective of the Chinese healthcare system using the Partitioned Survival model (PartSA model) and Dynamic Markov model (Markov model) were constructed.

## Materials and methods

### Population and intervention

Clinical information is derived from the most recent data from the IMpower110 study. Patients aged 18 years and older, histologically diagnosed with stage IV NSCLC (UICC/AICC 7th edition), epidermal growth factor receptor (EGFR) and anaplastic lymphoma kinase (ALK) wild type, and no prior treatment for NSCLC; OS benefit was seen only in patients with high PD-L1 expression (PD-L1 expressing tumor cells 50% or more), so only they were included in the study, 107 patients in the atezolizumab group and 98 patients in the chemotherapy group, respectively Drug doses were as follows: The experimental group received 1,200 mg atezolizumab intravenously every 3 weeks; non-squamous cell carcinoma patients in the control group received cisplatin 75 mg/m^2^ + pemetrexed 500 mg/m^2^ or AUC6 carboplatin + pemetrexed 500 mg/m^2^ for 4 to 6 cycles (calculated as 5 cycles), followed by pemetrexed maintenance therapy; patients with squamous cell carcinoma in the control group received cisplatin 75 mg/m^2^ + gemcitabine 1,250 mg/m^2^ or AUC5 carboplatin + gemcitabine 1,000 mg/m^2^ for 4 to 6 cycles (calculated as 5 cycles), followed by best supportive care. Gemcitabine was used twice per cycle (12, 15).

The mean body surface area was calculated using body data from The Report on Nutrition and Chronic Diseases of Chinese Residents (2010) (16) and the IMpower110 trial, and the drug doses were calculated. The probability of receiving cisplatin and carboplatin was assumed to be equal, since 69.7% of the patients had squamous cell carcinoma and 30.3% had non-squamous cell carcinoma.

Treatment after disease progression was based on the patient’s condition. Secondary therapy was assumed, as shown in **Supplementary Table 1**, based on drugs whose use exceeded 5% in the IMpower110 study. Nivolumab was used once every 2 weeks.

### Model structure

**Figure 1:**
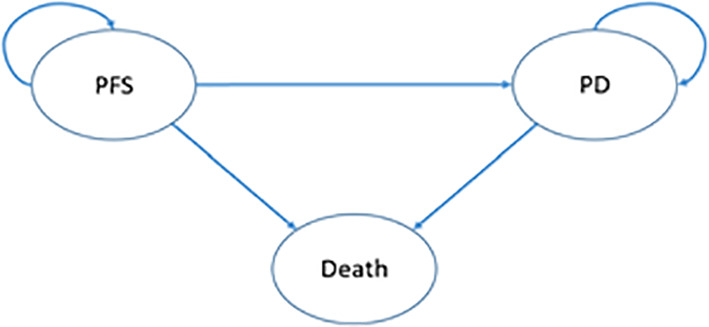
Model structure.

PartSA and Markov models were constructed using TreeageProHealthcare2022 software. Patients were divided into three states: progression-free survival (PFS), progression disease (PD), and death (Death) (according to the Response Evaluation Criteria in Solid Tumors version 1.1). All simulated patients started in the PFS state, progressed through the Healthy state, and finally ended in the Death state (**Figure 1**). In the Markov model, the time-dependent transition probabilities between states were calculated with survival curve parameters, and the probability of natural mortality was assumed to be the probability from PFS to death, with a half-cycle correction; in the ParSA model, the proportion of patients in different states was calculated in the area divided by the PFS and OS curves. The treatment cycle was assumed to be 3 weeks, in accordance with the treatment schedule. The horizon of the simulation study was 10 years (170 cycles, during which most patients died).

### Clinical data

Digital points were obtained from the OS and PFS curves reported in IMpower110 with GetData Graph Digitizer software, and individual data were reconstructed with Stata software. The median survival times of the reconstructed survival curves were compared with the original KM curves in **Supplementary Table 2**. As shown in **Supplementary Table 3**, we parameterized the reconstructed data using four survival distributions (Exponential, Weibull, Loglogistic, and lognormal) and finally evaluated the parametric survival distribution using the Akaike information criterion (AIC) and Bayesian information criterion (BIC). Finally, a lognormal distribution was chosen to fit the OS and PFS of the atezolizumab group and the OS of the chemotherapy group, resulting in the following survival function:


$$S(t)=1-\phi (\frac{\ln(t)-\mu }{\sigma })$$

The PFS of the chemotherapy group was fitted with a logistic distribution, and a survival function was calculated:


$$S(t)=\frac{1}{1+\lambda t^{Y}}$$

**Figure 2:**
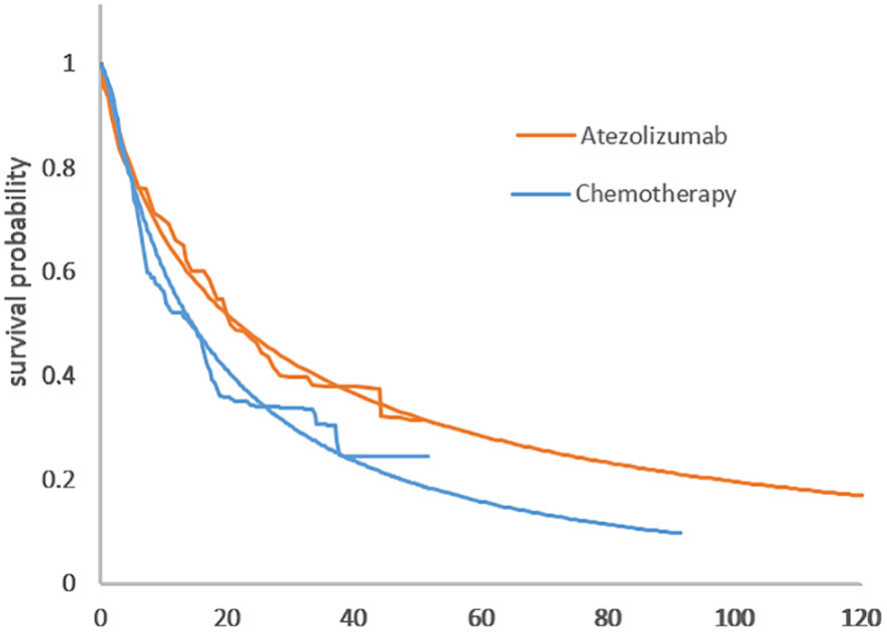
**(A)** Comparison of the adopted fitting curves and the KM survival curve of IMpower110. **(A)** PFS curve. **(B)** Comparison of KM survival curves with the fitting curve employed in IMpower110. **(B)** OS curve.

The distribution parameters of the four survival curves are estimated in **Table 1** and the survival curves are in **Figure 2**.

**Table 1:** Survival model parameters fitting to PFS and OS.

Group	Model	Parameter
OS of Atezolizumab	lognormal	μ=3.075809,σ=1.794862
OS of Chemotherapy	lognormal	μ=2.67934,σ=1.413161
PFS of Atezolizumab	lognormal	μ=2.187744,σ=1.583663
PFS of Chemotherapy	loglogistic	λ=0.06143,γ=1.70390

### Cost and utility estimate

Only direct medical costs were considered, including drug acquisition costs, side effect costs, IHC costs, best supportive care costs, and follow-up costs. We used data from a Chinese drug information website (www.yaozh.com), treatment costs from the West China Hospital (WCH) of Sichuan University in 2021, and relevant literature. Drug costs were calculated by multiplying the unit cost of a drug by the number of drugs used; if one drug was not used up in one treatment, it was considered wasted. The health utility values were derived from a 2018 study by Chinese scholars (17) focusing on advanced NSCLC using Euro-QoL-5dimensions (EQ-5D). The model also included the diseconomies of side effects. A discount rate of 5% per annum was used. ICER was calculated as a measure of cost-effectiveness in accordance with the China Pharmacoeconomic Evaluation Guidelines (2020) (18); $34,928.54 (September 2022 exchange rate, $1 = RMB6.955), three times the per capita gross domestic product (GDP) of China in 2021, was used as the willingness-to-pay (WTP). All parameter information is presented in **Table 2**.

**Table 2:** Model parameters: base-line values, ranges, and distributions for sensitivity analyses.

Variables	Baseline Value	Range	Distribution	Reference
Drug cost, US$/per cycle				
Atezolizumab	4716.03	3772.83 to 4716.03	Gamma	yaozh.com
Pembrolizumab	5152.55	4122.04 to 5152.55	Gamma	yaozh.com
Nivolumab(14days)	2644.75	2127.97 to 2644.75	Gamma	yaozh.com
Cisplatin	18.86	15.09 to 22.63	Gamma	WCH
Carboplatin(Weighted average)	33.59	26.87 to 40.30	Gamma	WCH
Pemetrexed	786.72	629.38 to 944.07	Gamma	WCH
Gemcitabine(Weighted average)	126.89	101.51 to 152.26	Gamma	WCH
Taxol	96.68	77.34 to 116.01	Gamma	WCH
Docetaxel	256.36	205.09 to 307.63	Gamma	WCH
Best supportive care	446.45	357.16 to 535.74	Gamma	WCH
AEs cost, US$				
Anemia	444.70	355.76 to 533.64	Gamma	WCH
Neutropenia	647.70	518.16 to 777.25	Gamma	WCH
Thrombocytopenia	1665.00	1332.00 to 1998.00	Gamma	WCH
Follow-up cost,US$/per cycle				
Register	2.01	1.61 to 2.42	Gamma	WCH l
Injection	0.86	0.69 to 1.04	Gamma	WCH
CT	158.16	126.53 to 189.79	Gamma	WCH
Blood routine tests	2.16	1.73 to 2.59	Gamma	WCH
Biochemical test	38.82	31.06 to 46.59	Gamma	WCH
Blood coagulation	6.33	5.06 to 7.59	Gamma	WCH
Utility				
PFS	0.856	0.8132 to 0.8988	Beta	(17)
PD	0.768	0.7296 to 0.8064	Beta	(17)
AEs disutility				
Anemia	−0.073	−0.058 to −0.088	Beta	(19)
Neutropenia	−0.650	−0.520 to −0.780	Beta	(19)
Thrombocytopenia	−0.460	−0.368 to −0.552	Beta	(19)
AEs incidence				
Anemia(Atezolizumab)	0.017	0.0153 to 0.0187	Beta	(12)
Neutropenia(Atezolizumab)	0.007	0.0063 to 0.0077	Beta	(12)
Thrombocytopenia(Atezolizumab)	0.003	0.0027 to 0.0033	Beta	(12)
Anemia(Chemotherapy)	0.190	0.1710 to 0.2090	Beta	(12)
Neutropenia(Chemotherapy)	0.175	0.1575 to 0.1925	Beta	(12)
Thrombocytopenia(Chemotherapy)	0.076	0.0684 to 0.0836	Beta	(12)
Others				
Immunohistochemistry(IHC) cost	115.03	92.02 to 138.03	Gamma	WCH
Discount rate	0.05	0.00 to 0.08	–	

### Sensitivity and scenario analysis

One-way sensitivity analysis (OSA) and probabilistic sensitivity analysis (PSA) were used to test the uncertainty of the model; OSA assumed that the prices of atezolizumab, pembrolizumab, and nivolumab could only be reduced and used ±20% of the base case value or 95% of confidence intervals were used as the basis for the range of uncertainty for each parameter. The results were displayed on a tornado diagram; PSA ran a Monte Carlo simulation with 1,000 iterations to generate a cost-effectiveness acceptability curve and a cost-effectiveness scatterplot to represent the results.

To improve the cost-effectiveness of covered drugs, the Chinese government has adopted a drug price negotiation mechanism, directly reduced the reimbursement price of medical insurance, discounted the entire course of treatment, and implemented a patient assistance program (PAP) (20). In the scenario analysis, it was assumed that half of the patients met the PAP requirements.

In China, there was a large gap between rich and poor in different provinces: the highest per capita GDP for provinces and cities in 2021 was $79,358.73 (Beijing) and the lowest was $17,704.96 (Gansu), a difference of 4.48 times. Assuming a WTP of three times the national per capita GDP may ignore regional differences. In the scenario analysis, we estimated the cost-effectiveness price of atezolizumab when the WTP was set at 1 and 3 times the GDP per capita for each region.

## Results

### Base-case analysis

In the PartSA model, the total cost for the atezolizumab group was $145,038, yielding 2.92 LYs and 2.39 QALYs, while the total cost for the chemotherapy group was $69,803, yielding 2.12 LYs and 1.65 QALYs. The ICER for atezolizumab versus chemotherapy was $102,424.83/QALY; in the Markov model, the ICER was $104,806.71/QALY. Atezolizumab was not cost-effective at the WTP threshold of three times per capita GDP; when PAP was available, ICERs for atezolizumab and chemotherapy in the PartSA and Markov models were $49,213.93/QALY and $54,874.23/QALY, respectively. QALY; although PAP reduced costs significantly, atezolizumab was still not cost-effective at the WTP threshold (**Table 3**).

**Table 3:** Base-case results.

Strategies and Scenarios	PartSA Model				Markov Model			
	Total cost	LYs	QALYs	ICER($/AQLY)	Total cost	LYs	QALYs	ICER($/AQLY)
Without PAP								
Atezolizumab	145,038	2.92	2.39	102,424.83	135,044	2.76	2.25	104,806.71
Chemotherapy	69,803	2.12	1.65		59,365	2.09	1.52	
With PAP								
Atezolizumab	105,953	2.92	2.39	49,213.93	98,988	2.76	2.25	54,874.23
Chemotherapy	69,803	2.12	1.65		59,365	2.09	1.52	

### Sensitivity analysis

**Figure 3:**
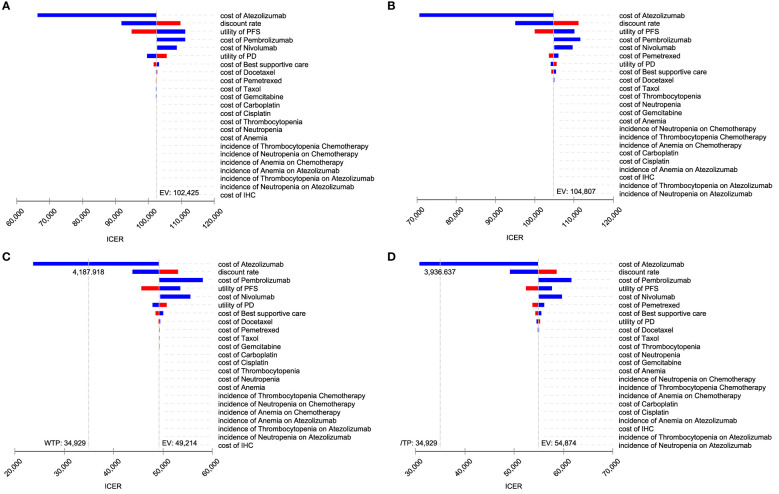
Toronto diagram of one-way sensitivity analysis. **(A)** PartSA mode. **(B)** Markov model. **(C)** PartSA model when PAP is available. **(D)** Markov model when PAP is available.

The Tornado diagram shows that the crucial parameters impacting ICER were similar in the two models. The cost of atezolizumab, the utility value of PFS, and the discount rate had a pronounced impact on ICER. For a range of parameters, ICER was higher than WTP, making atezolizumab not cost-effective for chemotherapy (**Figures 3A, B**) In the presence of PAP, the cost of atezolizumab, the discount rate, the cost of pembrolizumab, and the utility value for PFS had a significant impact on ICER. When the price of atezolizumab was reduced to $4,187.92 (PartSA model) or $3936.64 (Markov model), atezolizumab was cost-effective at the WTP threshold (**Figures 3C, D**).

**Figure 4:**
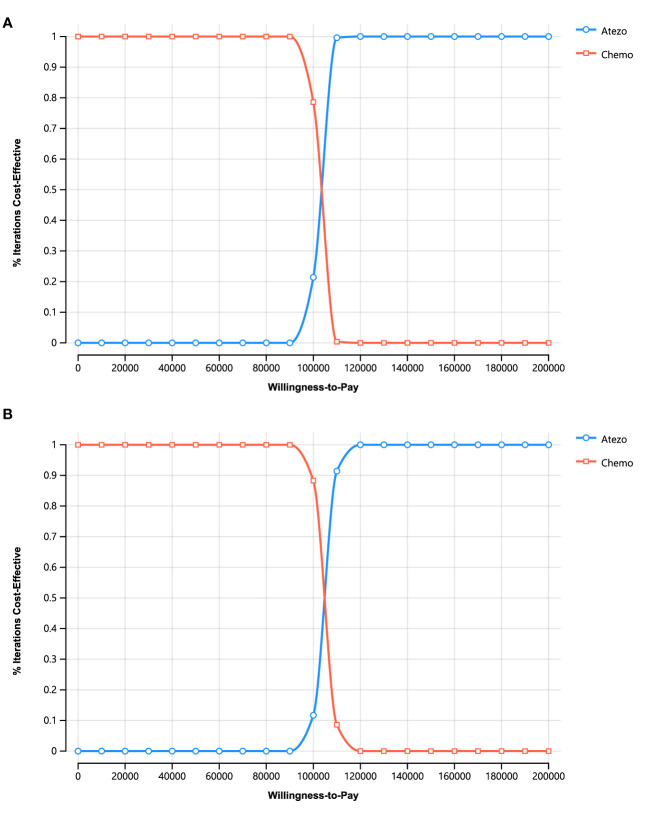
Cost-effectiveness acceptability curves for stochastic analysis. **(A)** PartSA model. **(B)** Markov Model.

For PSA, the cost-effectiveness scatterplots (**Supplementary Figure 1**) and cost-effectiveness acceptability curves (**Figure 4**) indicate that atezolizumab provides more QALYs at a higher cost At the WTP threshold, the probability that atezolizumab is cost-effective is 0% At the WTP threshold of greater than $102,424.83 (PartSA model) or $104,806.71 (Markov model), the probability that atezolizumab monotherapy was more cost-effective than chemotherapy increased.

### Scenario analysis

In the PartSA model, the cost-effectiveness price of atezolizumab nationwide was $2,347.72 and $2,955.19 when WTP was 1x and 3x per capita GDP, respectively. In Beijing, they were $2,734.08 and $4,114.29; in Gansu, they were $2,197.94 and $2,505.87. With PAP, the national cost-effectiveness prices for atezolizumab were $3,327.03 and $4,187.92; in Gansu, they were $3,114.78 and $3,551.14 dollars. In Beijing, the original price of atezolizumab was cost-effective versus chemotherapy when the WTP was three times the GDP per capita (**Table 4**).

**Table 4:** Cost-effective price of atezolizumab in different regions.

Scenarios and WTP	PartSA Model			Markov Model		
	Beijing	China	Gansu	Beijing	China	Gansu
Without PAP						
WTP=GDP per capita	2734.08	2347.72	2197.94	2550.27	2140.9	1982.21
WTP=three times GDP per capita	4114.29	2955.19	2505.87	4012.63	2784.54	2308.47
With PAP						
WTP=GDP per capita	3874.56	3327.03	3114.78	3605.42	3026.68	2802.34
WTP=three times GDP per capita	–	4187.92	3551.14	–	3936.64	3263.57

## Discussion

Breakthroughs in tumor immunotherapy, for which the Nobel Prize in Physiology or Medicine was awarded, offer hope for the survival of patients with malignant tumors. As immune checkpoint inhibitors become the new standard of care for various tumor therapies, the very high cost of these drugs poses a significant challenge to the healthcare system. The introduction of lung cancer immunotherapy was accompanied by improved survival rates and increased expenditures (21). Therefore, increased pharmaceutical spending is in direct competition with other health and social spending, and therefore, an econometric study should be conducted that carefully balances the awarding of innovation with ensuring affordability. This study is the first cost-effectiveness evaluation comparing atezolizumab monotherapy to platinum-based chemotherapy, based on the most recent data from IMpower110. The PartSA and Markov models show that first-line atezolizumab monotherapy for advanced NSCLC is not cost-effective versus chemotherapy. When PAP became available, ICER decreased significantly, but was still not cost-effective OSA showed that the cost of atezolizumab, PFS utility, and discount rate were the most influential factors; when the WTP threshold exceeded $102,424.83 (PartSA model) or above $104,806.71 (Markov model), the probability that atezolizumab monotherapy was more cost-effective than chemotherapy increased.

Several econometric studies based on preliminary data from the IMpower110 study (13, 14, 22) are shown in **Table 5**; The WTP in the United States is much higher than the WTP in China, indicating that atezolizumab is likely to be cost-effective from a U.S. perspective. In addition, atezolizumab was rated as cost-effective in areas with a high level of economic development, such as Beijing, China. A study by Shen Li et al. (23) focused on atezolizumab plus chemotherapy in 2022 from the perspective of the US healthcare system, with an ICER of $130,804.59/QALY. Atezolizumab combination therapy as first-line treatment was similar to atezolizumab monotherapy. Although atezolizumab is not cost-effective at this time, its clinical benefits are significant: in the IMpower110 trial, the duration of response (DoR) was up to 38.9 months, the longest of any current immunologic agent, validating its long-tailing effect. If remission is achieved with atezolizumab, there is the potential for long-term benefit. The cost of atezolizumab was the most influential parameter for cost-effectiveness. The study showed that atezolizumab becomes cost-effective when the cost of atezolizumab falls below $2308.47. In 2015, the Chinese government initiated national healthcare negotiations to discuss drug prices for expensive drugs such as cancer drugs and orphan drugs, expanding reimbursement coverage and lowering drug prices. The government agreed to expand reimbursement coverage and reduce drug prices. However, imported immunotherapy drugs such as atezolizumab and pembrolizumab will no longer be eligible for national healthcare reimbursement in China, and their use in clinical practice will be limited. This study provides evidence of the fair price of atezolizumab in China and will help policy-making departments in their economic strategies. Patient assistance programs are also an effective means of improving the economics of atezolizumab, reducing ICERs by about 50%; Guoqiang Liu’s study (14) also mentions patient assistance programs, which can reduce ICERs by about 60%-65%. Another advantage of patient assistance programs is that they can target patients who are truly in financial need rather than a general decline in drug prices.

**Table 5:** Comparison of published studies.

Study	Perspective	Model	Cost	WTP	ICER	Conclusion	Scenario analysis
(21)Ye peng 2021	US payer perspective	Markov model	Drug cost; AE cost; Subsequent Treatment cost; Administration cost	$100,000-150,000/QALY	$170,730/QALY	not to be cost-effective	None
(14)Guoqiang liu 2021	Chinese health sector perspective	Markov model	Drug cost; AE cost; Supportive care cost; Follow-up cost; Terminal cost	1-3times of GDP	High PD-L1 $123,778.60/QALY; High-or-intermedia PD-L1 $142,827.19/QALY; Any PD-L1 $168,902.66/QALY	not to be cost-effective	With PAP
(13)Shuqiao Cheng 2021	US and Chinese payer perspective	Partitioned survival model	Drug cost; AE cost; Administration cost; IHC cost ; Follow-up cost; Best supportive cost; Terminal cost	US $100,000-150,000/QALY; China 1-3times of GDP	China $78,936/QALY; US $123,424/QALY	US to be cost-effective; China not to be cost-effective	None
This study	Chinese health sector perspective	Markov model/ Partitioned survival model	Drug costs; AE costs; IHC costs; Best supportive care costs; Follow-up costs; Subsequent Treatment cost	1-3times of GDP	PartSA model $102,424.83/QALY; Markov model $104,806.71/QALY.	not to be cost-effective	With PAP; with different regions

WTP willingness-to-pay, ICER incremental cost-effectiveness ratios, QALY quality-adjusted life years, PAP Patient Assistance Program.

A controversial aspect of cost-effectiveness evaluation was the choice of a simulation model (24–26). Economic models have uncertainties, and sensitivity analyses were used to address parameter uncertainties, but Partitioned Survival and Markov models were often used, where structural uncertainties due to alternative models were generally not addressed. In the traditional Markov model, additional assumptions were often made, such as whether persons in PFS and PD states were allowed to transition to a mortality state; in the Partitioned Survival model, survival data reported in clinical trials were used to avoid estimating transition probabilities. In a study by McEwan et al. (27), they found that the PartSA model most accurately reproduced observed survival outcomes. However, Coly et al. (28) found that the PartSA model has an inherent bias in favor of treatment induced by disease progression. Rui and Goeree et al. (24, 29) believe that results such as ICER obtained with the PartSA and Markov models are nearly identical. Smare and Williams et al. (25, 26) pointed out that the prediction results of the two models differ. Although they ultimately reached the same conclusion, this study showed that there are differences between the two models based on empirical evidence. Therefore, the use of multiple models for economic evaluation may be a way to reach robust conclusions. This analysis had several limitations worth considering. First, only patients with PD-L1 expression ≥50% in the IMpower110 trial were included because they were the most efficient, but the characteristics of all advanced NSCLC patients cannot be generalized. Second, the utility values were obtained from the literature and may not be representative of real patients. Third, the support and follow-up costs in this study were based on price estimates for relevant treatment and laboratory items in Chinese hospitals, while the second line of treatment was derived from clinical trials. Because actual patient situations and treatment costs may differ, a sensitivity analysis was performed, but the costs in this part of the study do not affect the conclusions. Fourth, to make the model easier to understand and compute, several assumptions were made to simulate and simplify real-world situations, including parametric fitting and extrapolation of survival curves, the assumption that patients have three states (PFS, PD, and death), and in the Markov model, the probability that a patient moves from the PFS state to the death state is natural death.

## Conclusion

First-line monotherapy with atezolizumab for patients with PD-L1 high-expressing EGFR and ALK wild-type advanced NSCLC was estimated to be less cost-effective than chemotherapy in terms of the Chinese healthcare system; offering PAP increased the likelihood that atezolizumab would be cost-effective. In some areas of China with higher levels of economic development, atezolizumab was likely to be cost-effective. To improve the cost-effectiveness of atezolizumab, drug prices would need to be reduced.

## Data availability statement

The original contributions presented in the study are included in the article/**Supplementary Material**. Further inquiries can be directed to the corresponding authors.

## Author contributions

CZ: Literature search, study selection, analysis of the two model, writing -review & editing. YL: Analysis of the two model, writing - original draft. JT: Writing-review & editing. PT: Formulation or evolution of overarching research aims and writing-review & editing. WL: Ideas, formulation or evolution of overarching research aims, supervision and project administration. All authors contributed to the article and approved the submitted version.
