# Supplementary material for: Cost-effectiveness evaluation based on two models of first-line atezolizumab monotherapy and chemotherapy for advanced non-small cell lung cancer with high-PDL1 expression
Source: Front Oncol. 2023 Mar 14;13:1093469. doi: 10.3389/fonc.2023.1093469 (PMC10043365; doi:10.3389/fonc.2023.1093469)
Supplement: Supplementary file 1 [file DataSheet_1.docx]

Supplementary Material Presentation

**Supplementary Table 1** | Second-line treatment assumption

| Treatment | Rate（%） | |
| --- | --- | --- |
|  | Atezolizumab | Chemotheropy |
| Carboplatin | 19.6 | 5.1 |
| Pemetrexed | 12.1 | - |
| Gemcitabine | 9.4 | - |
| Taxol | 8.4 | - |
| Cisplatin | 7.2 | - |
| Docetaxel | 6.5 | 13.3 |
| Pembrolizumab | - | 19.4 |
| Nivolumab | - | 17.9 |
| Best suppotive care | 36.8 | 44.3 |

**Supplementary Table 2** | The median survival time comparison of the reconstructed survival curve and original KM curve in IMpower110 trial

| Group | Reconstructed(month) | Original(month) |
| --- | --- | --- |
| OS of Atezolizumab | 20.20052 | 20.2 |
| OS of Chemotherapy | 14.69683 | 14.7 |
| PFS of Atezolizumab | 8.22642 | 8.2 |
| PFS of Chemotherapy | 5.09852 | 5.0 |

**Supplementary Table 3** | Summary of statistical goodness-of-fit of reconstructed survival curve

| Group | Model | exponential | weibull | loglogistic | lognormal |
| --- | --- | --- | --- | --- | --- |
| OS of Atezolizumab | AIC | 337.0264 | 333.1187 | 329.8031 | 328.6794 |
|  | BIC | 339.6993 | 338.4643 | 335.1487 | 334.025 |
| OS of Chemotherapy | AIC | 297.3628 | 297.4969 | 288.6452 | 286.1551 |
|  | BIC | 299.9478 | 302.6668 | 293.8151 | 291.325 |
| PFS of Atezolizumab | AIC | 389.4144 | 378.4611 | 365.3031 | 363.0774 |
|  | BIC | 392.0872 | 383.8068 | 370.6488 | 368.423 |
| PFS of Chemotherapy | AIC | 292.6219 | 294.2565 | 280.1847 | 281.5601 |
|  | BIC | 295.2068 | 299.4265 | 285.3546 | 286.7301 |

**A**  **B**


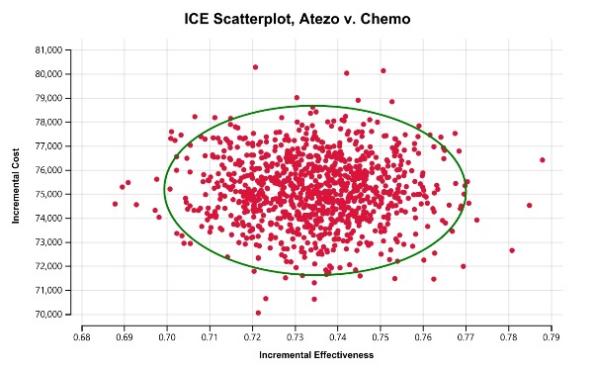

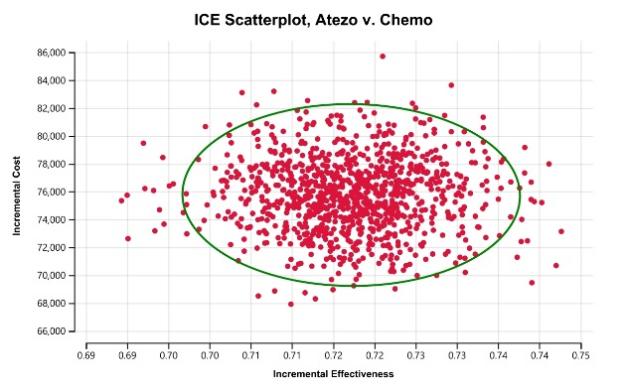


**Supplementary Figure 1** | cost-effectiveness scatter chart of probabilities analyses. **(A)** PartSA model **(B)** Markov model

Reference

1. Shen Y, Wu B, Wang X, Zhu J. Health State Utilities in Patients with Advanced Non-Small-Cell Lung Cancer in China. *J Comp Eff Res* (2018) 7(5):443-52. Epub 20180518. doi: 10.2217/cer-2017-0069.

2. Nafees B, Lloyd AJ, Dewilde S, Rajan N, Lorenzo M. Health State Utilities in Non-Small Cell Lung Cancer: An International Study. *Asia Pac J Clin Oncol* (2017) 13(5):e195-e203. Epub 20160317. doi: 10.1111/ajco.12477.

3. Jassem J, de Marinis F, Giaccone G, Vergnenegre A, Barrios CH, Morise M, et al. Updated Overall Survival Analysis from Impower110: Atezolizumab Versus Platinum-Based Chemotherapy in Treatment-Naive Programmed Death-Ligand 1-Selected Nsclc. *J Thorac Oncol* (2021) 16(11):1872-82. Epub 20210712. doi: 10.1016/j.jtho.2021.06.019.
